# Identification of CKAP2 as a Potential Target for Prevention of Gastric Cancer Progression: A Multi-Omics Study

**DOI:** 10.3390/ijms26041557

**Published:** 2025-02-12

**Authors:** Xueyi Liu, Wenyu Zhang, Hui Wang, Wulin Yang

**Affiliations:** 1 Anhui Province Key Laboratory of Medical Physics and Technology, Institute of Health and Medical Technology, Hefei Institutes of Physical Science, Chinese Academy of Sciences, Hefei 230031, China; lxy22168090@mail.ustc.edu.cn (X.L.); quii1210@163.com (W.Z.); 2 Science Island Branch, Graduate School of University of Science and Technology of China, Hefei 230031, China; 3 Laboratory of Molecular Genetics of Aging & Tumor, Medicine School, Kunming University of Science and Technology, Kunming 650500, China; huiwang266@163.com

**Keywords:** gastric cancer, aging, CKAP2

## Abstract

Gastric cancer (GC) ranks as one of the most prevalent malignant tumors globally. The subtle manifestation of its early-stage symptoms often results in many GC patients being diagnosed at a late or advanced stage, thereby posing significant obstacles to the effectiveness of chemotherapy treatments. Therefore, identifying early biomarkers for GC is crucial. In recent years, an increasing number of studies have highlighted the pivotal role that aging plays in the progression of cancer. Among the various proteins involved, Cytoskeleton-associated protein 2 (CKAP2) emerges as a crucial player in controlling cell proliferation, regulating mitosis and cell division, and exerting a significant influence on the aging process. We employed a bioinformatics approach to assess the causal association between aging-related genes and GC and explore the potential significance of CKAP2 in GC by analyzing data sourced from various repositories, including Genotype Tissue Expression (GTEx), GWAS Catalog, The Database of Cell Senescence Genes (CellAge), The Cancer Genome Atlas (TCGA), Gene Expression Omnibus (GEO), Human Protein Atlas (HPA), and the Comparative Toxicology Genome Database (CTD). Our research summarized the causal relationship between CKAP2 expression and the development risk of GC, differential expression in GC, the relationship with the prognosis of GC, genetic correlation, functional analysis, and immune cell infiltration, and explored the interaction of CKAP2 and chemical substances. The findings revealed that an elevation in CKAP2 expression correlated with a reduced likelihood of developing GC. There was a significant difference in the expression of CKAP2 between GC and normal patients. Specifically, there was higher expression in GC compared to normal patients. In addition, CKAP2 has been proven to have diagnostic value in GC, and elevated levels of CKAP2 expression are indicative of a more favorable prognosis. Immune infiltration analysis revealed the relationship between CKAP2 and tumor immune microenvironment, while the Comparative Toxicology Genome Database (CTD) identified a small molecule compound that may target CKAP2. In summary, through comprehensive multivariate analyses, we identified and validated the potential role that CKAP2 may play in GC. Therefore, CKAP2 shows potential as an indicator for both the diagnosis and prognosis of GC, making it worthy of further clinical investigation.

## 1. Introduction

Gastric cancer (GC) poses a formidable challenge to global health, standing as the fifth most common cancer in incidence and the fourth leading cause of cancer-related deaths worldwide [1]. In 2020 alone, over 1 million fresh instances of GC were documented, leading to approximately 769,000 deaths [2]. The early symptoms of GC are often subtle, leading to the diagnosis of the majority of patients at advanced stages of the disease, for which standard treatments are largely ineffective [3,4]. The Tumor, Node, and Metastasis (TNM) guidelines serve as a comprehensive staging system for describing and classifying cancers, widely utilized to assess tumor severity and predict prognosis [5]. Nevertheless, given the considerable heterogeneity in disease progression, the TNM staging system alone may not offer an entirely dependable prognostic tool [6,7]. Therefore, the identification of biomarkers for early diagnosis and effective treatment of GC holds the utmost importance.

Accumulating evidence indicates that the development of GC encompasses numerous biological processes, including gene mutations [8], microsatellite instability [9], inflammation [10], oxidative stress [11], DNA methylation [12], and Epithelial-Mesenchymal Transition (EMT) [13]. Among these factors, aging emerges as a pivotal player in the onset and progression of GC. Aging is a multifaceted and intricate process that results in various organ impairments and a spectrum of age-associated disorders [14,15], such as neurodegenerative disorders, diabetes, idiopathic pulmonary fibrosis, etc. [16]. DNA damage serves as a key instigator of the aging process, and the efficacy of DNA repair is a crucial determinant in this process [17]. Furthermore, impaired DNA repair accelerates the development of various age-related disorders [18]. Aging is associated with various pathways involved in GC, but its exact mechanisms remain unclear. Therefore, additional research endeavors are imperative to gain a more profound comprehension of the role that aging plays in the progression of GC.

In this work, we evaluated the causal association between aging-related genes and GC by Genotype Tissue Expression (GTEx) and GWAS Catalog data. Expression of cytoskeleton-associated protein 2 (CKAP2) and p21-activated kinase 4 (PAK4) in GC was analyzed using TCGA and GEO data. Furthermore, we analyzed the expression of CKAP2 and PAK4 in relation to GC prognosis, genetic correlation, functional analysis, immune cell infiltration, and the interaction of CKAP2 and chemical substances. The objective of this study was to elucidate the potential functions and regulatory mechanisms of CKAP2 in the pathogenesis and prognosis of GC. This further suggests that CKAP2 holds promise as both a biomarker and therapeutic target for GC.

## 2. Results

### 2.1. Identification of Aging-Related Genes Associated with GC

**Figure 1:**
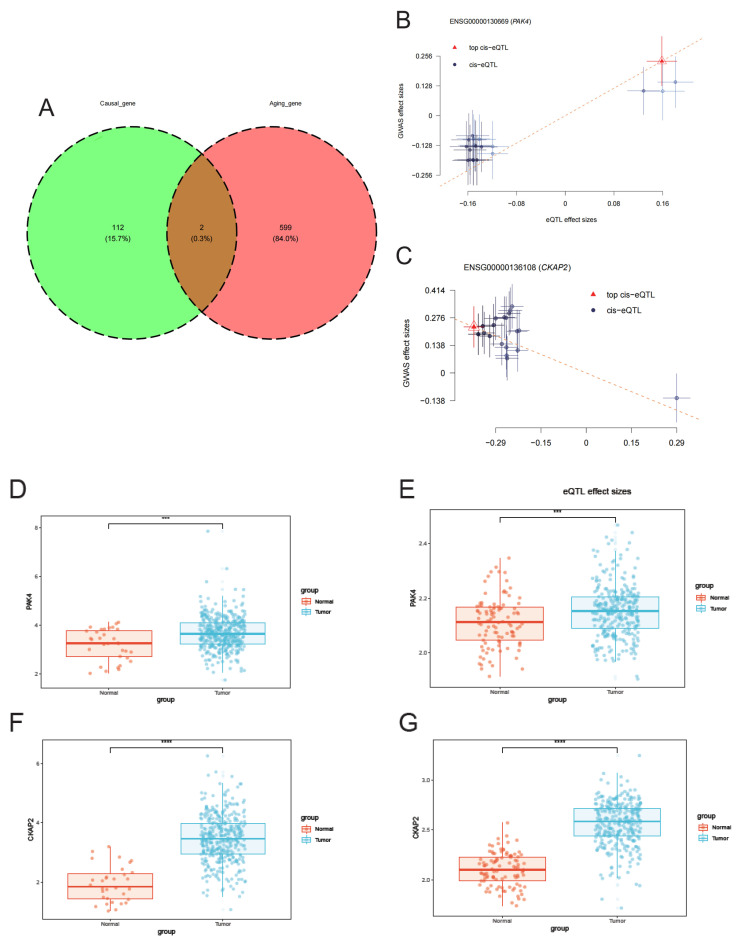

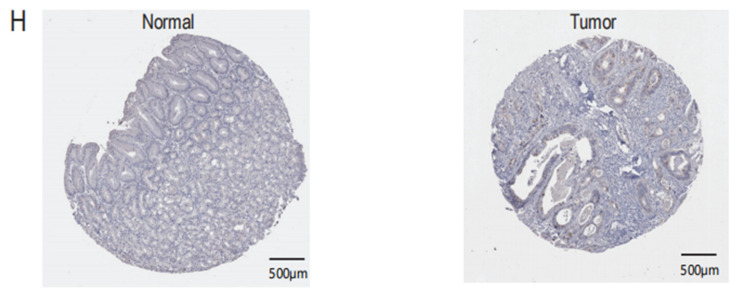
Identifying aging-related genes in GC. (**A**) A Venn diagram illustrating the overlap between causally related GC genes and aging-associated genes. (**B**,**C**) Causal association between PAK4, CKAP4, and GC. (**D**,**F**) PAK4 and CKAP2 expression in tumor and normal tissues from the TCGA dataset. (*** *p* < 0.001, **** *p* < 0.0001) (**E**,**G**) PAK4 and CKAP2 expression in tumor and normal tissues from the GEO dataset. (**H**) Expression of CKAP2 protein in normal and GC tissues.

We retrieved expression Quantitative Trait Locus (eQTL) data for stomach tissues from the GTEx database and genome-wide association study for subsequent Summary-data-based Mendelian Randomization (SMR) analysis. This comprehensive analysis ultimately led to the identification of 114 genes linked with GC, as listed in Table S1. Next, we intersected these risk-related genes with aging-related genes pertinent to GC, ultimately pinpointing two aging-related GC genes: PAK4 (p-SMR = 0.036, p-HEIDI = 0.409) and CKAP2 (p-SMR = 0.027, p-HEIDI = 0.316) (Figure 1A). Specifically, elevated expression of PAK4 was associated with a raised GC risk, whereas increased expression of CKAP2 correlated with a decreased risk of GC (Figure 1B,C). To further verify these findings, we analyzed the expression of PAK4 and CKAP2 in datasets from TCGA, GEO cohort, and immunohistochemical results of GC patients compared to normal controls. The results consistently demonstrated that both PAK4 and CKAP2 were significantly upregulated in GC tissues in comparison with normal tissues (Figure 1D–H).

### 2.2. Prognostic Value of CKAP2 and PAK4 Expression

**Figure 2:**
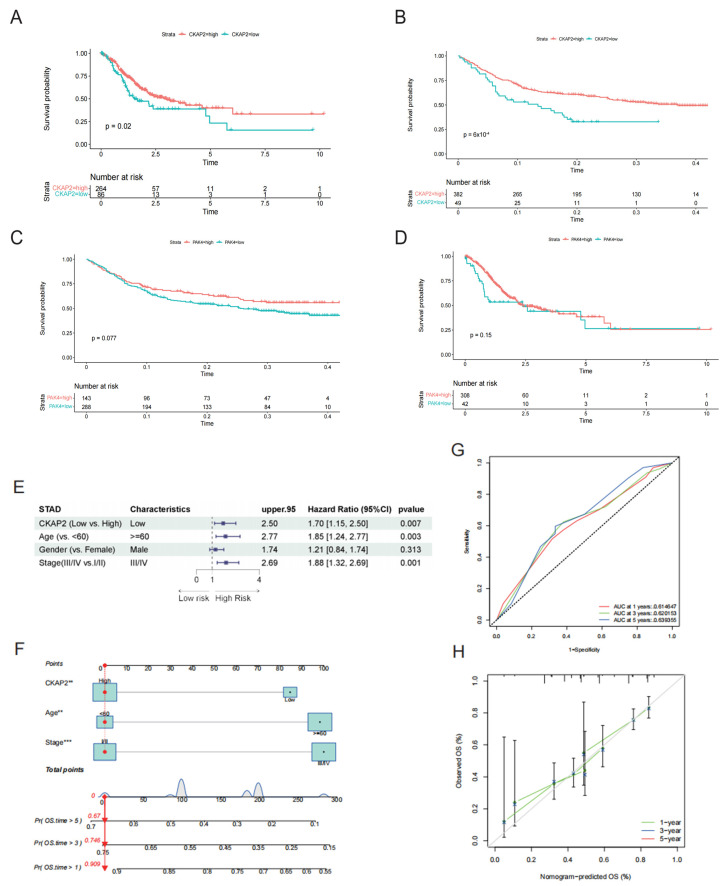
Prognostic Significance of CKAP2 and PAK4. (**A**,**C**) Survival Analysis in TCGA Cohort: Kaplan–Meier analysis revealed distinct survival outcomes for patients with high versus low expression levels of CKAP2 and PAK4, respectively, in the TCGA dataset. (**B**,**D**) Survival Analysis in GEO Dataset: Similarly, Kaplan–Meier analysis demonstrated differential survival rates based on high and low expression levels of CKAP2 and PAK4 in the GEO dataset. (**E**) Multivariate Cox Regression Analysis in TCGA Dataset: This analysis assessed the prognostic impact of CKAP2 expression levels (high vs. low) along with related clinical characteristics in patients from the TCGA dataset. (**F**) Nomogram of Prognostic Factors: A nomogram was constructed to visualize the combined effect of prognostic factors. (** *p* < 0.01, *** *p* < 0.001) (**G**) ROC Curve for Survival Prediction: The ROC curve was employed to evaluate the predictive accuracy for 1-, 3-, and 5-year survival rates in the TCGA dataset. (**H**) Calibration Curve for Survival Prediction: A calibration curve was utilized to assess the precision of 1-year and 3-year survival predictions.

Firstly, we examined the correlation between CKAP2 expression and the prognosis of GC patients. Our findings revealed that GC patients with high CKAP2 expression exhibited a longer survival duration (Figure 2A). To further substantiate this association, we confirmed the correlation between CKAP2 expression and survival rates of GC patients using an external cohort obtained from the GEO database, as depicted in Figure 2B. Furthermore, we examined the association between PAK4 expression and the prognosis of GC patients, with the results further corroborated by the GEO database. However, the results demonstrated that PAK4 expression was not significantly related to survival in GC patients (Figure 2C,D). Subsequently, we employed Multivariate Cox regression to investigate the association of CKAP2 expression, along with clinical characteristics such as age, gender, and clinical stage, with the prognosis of GC patients. Our analysis demonstrated that CKAP2 expression, age, and clinical stage were significantly linked to GC prognosis (Figure 2E).

To provide a clinical tool for prognosis, we constructed a nomogram incorporating these factors. Utilizing this nomogram, we were able to forecast the 1-year, 3-year, and 5-year overall survival rates for GC patients, taking into account factors such as age, clinical stage, and CKAP2 expression, as illustrated in Figure 2F. To evaluate the effectiveness and efficiency of this nomogram, we employed a time-varying ROC curve, revealing an area under the curve (AUC) for each of the 1-year, 3-year, and 5-year predictions that surpassed 0.6, as shown in Figure 2G. To further validate our model, we utilized calibration curves to assess its predictive accuracy. Notably, the 1-year and 3-year calibration curves demonstrated a close alignment with the 45-degree diagonal, indicating a strong agreement between the predicted and actual survival probabilities (Figure 2H). Additionally, we explored the relationship between CKAP2 expression and various clinical stages of GC, as presented in Figure S1.

### 2.3. Correlation Analysis and Functional Enrichment

**Figure 3:**
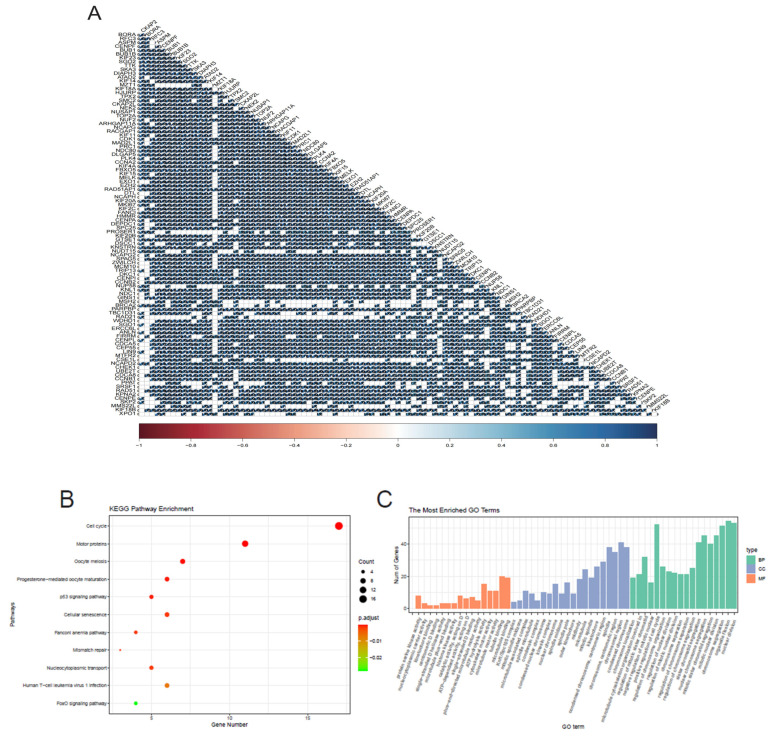
Correlation and Functional Insights. (**A**) Correlation Analysis: A Pearson correlation analysis was conducted to investigate the genes related to CKAP2 expression. (**B**) KEGG Enrichment Scatter Plot. (**C**) GO Enrichment Histogram.

We calculated the top 100 genes co-expressed with CKAP2 by Pearson correlation (Figure 3A). Based on these co-expressed genes, we conducted KEGG and GO function enrichment analyses (Figure 3B,C). These genes were primarily enriched in the cell cycle, motor proteins, oocyte meiosis, p53 signaling pathway, cellular senescence, positive regulation of the cell cycle, organelle fission, nuclear division, and chromosome segregation, according to KEGG and GO enrichment analysis.

### 2.4. Biological Functions Related to CKAP2

**Figure 4:**
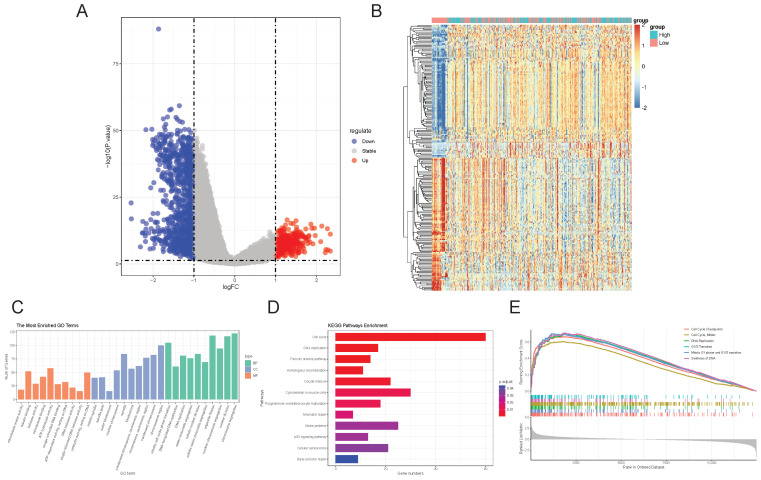
CKAP2-Associated Biological Functions. (**A**) Volcano Plot of DEGs: A volcano plot was used to visualize the differentially expressed genes (DEGs). (**B**) Heatmap of Top 200 DEGs: A heatmap was constructed to showcase the top 200 DEGs. (**C**–**E**) GO, KEGG Enrichment, and GSEA Plots.

Based on the observed correlation between CKAP2 expression and GC prognosis, we divided patients into high and low-expression groups using the median CKAP2 expression level as the cutoff. Subsequent differential expression analyses were performed to identify genes with significant up- or down-regulation (Figure 4A). A heatmap illustrating the top 200 differentially expressed genes is provided in Figure 4B. GO enrichment analysis revealed that these genes were enriched in processes such as chromosome segregation, organelle fission, mitotic cell cycle phase transition, nuclear division, and nuclear chromosome segregation (Figure 4C). Additionally, KEGG enrichment analysis showed that these genes were enriched in pathways such as the cell cycle, cytoskeleton in muscle cells, DNA replication, and the Fanconi anemia pathway (Figure 4D). GSEA enrichment analysis further demonstrated that activities related to cell cycle checkpoints, the cell cycle, mitosis, DNA replication, the G1/S transition, the mitotic G1 phase, and the G1/S transition and synthesis of DNA were upregulated in the high CKAP2 expression group (Figure 4E).

### 2.5. The Association Between CKAP2 Expression and Immunity

**Figure 5:**
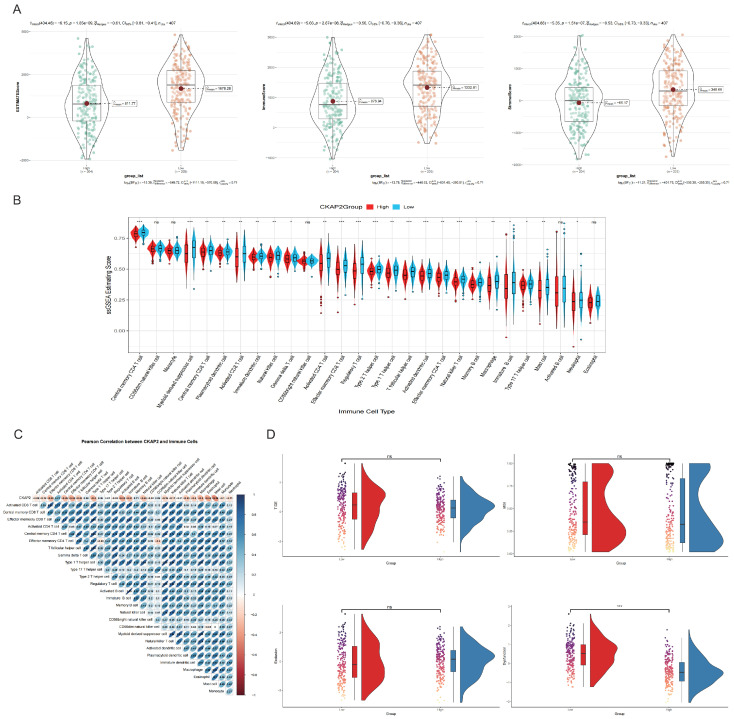
CKAP2 Expression and Its Association with Immunity. (**A**) Immune and Stromal Scores vs. CKAP2 Expression: This plot explores the relationship between CKAP2 expression and both immune and stromal scores. (**B**) Immune Cell Abundance and CKAP2 Expression: This section analyzes the correlation between CKAP2 expression and the abundance of various immune cell types. (* *p* < 0.05, ** *p* < 0.01, *** *p* < 0.001, and “ns” *p* > 0.05). (**C**) Pearson Correlation Analysis of CKAP2 and Immune Cells: A Pearson correlation analysis is conducted to quantify the relationship between CKAP2 expression and specific immune cell populations. (**D**) CKAP2 and Immune Checkpoints: This plot investigates the potential link between CKAP2 expression and immune checkpoint molecules.

The development of GC is intricately linked to its tumor microenvironment. Therefore, we delved into the relationship between CKAP2 expression and immune responses. Firstly, we examined the correlation between CKAP2 expression and immune and stromal scores, discovering that patients with elevated CKAP2 expression had lower scores in both categories (Figure 5A). Secondly, we applied ssGSEA to compare the immune cell abundances between the high and low CKAP2 expression groups, revealing significant differences in the populations of 23 immune cell types between these two cohorts (Figure 5B). Thirdly, we examined the correlation between CKAP2 and 28 immune cell types, finding that CKAP2 was linked to various immune cell subpopulations (Figure 5C). Lastly, we utilized the TIDE tool to delve into the mechanisms underlying tumor immune escape and to predict the responsiveness to immune checkpoint inhibitors (ICIs). Our results found that the low CKAP2 expression group displayed higher levels of immune dysfunction, whereas no significant changes were observed in terms of Exclusion, microsatellite instability (MSI), and TIDE scores (Figure 5D).

### 2.6. Annotation of Cell Type and Immune Infiltration Analysis

**Figure 6:**
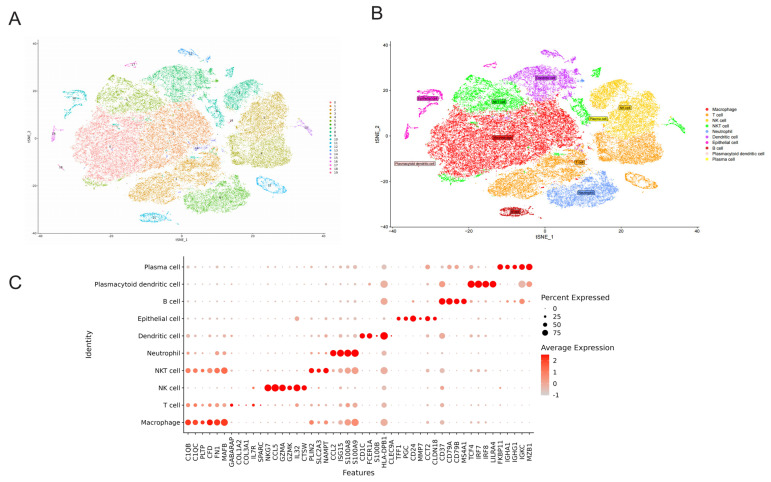

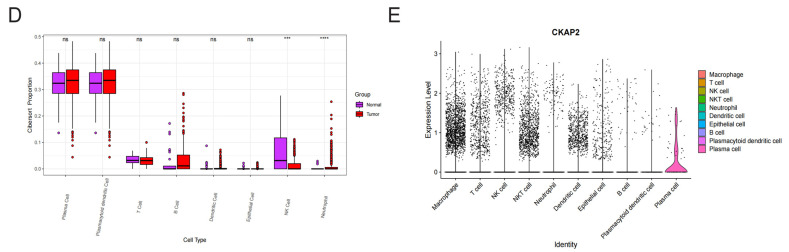
Cell Type Identification and Immune Infiltration Analysis. (**A**) Single-Cell TSNE Dimensionality Reduction. (**B**) Marker Gene Annotation Identifies Cell Types: Through marker gene annotations, ten distinct cell types were identified. (**C**) Marker Gene Bubble Map for Cell Types: A bubble map visually represents the marker genes associated with the ten identified cell types. (**D**) TCGA Cohort Annotation Using CIBERSORT: Eight cell types were annotated within the TCGA cohort utilizing the CIBERSORT algorithm. (“ns” *p* > 0.05, *** *p* < 0.001, and **** *p* < 0.0001). (**E**) CKAP2 Expression Across Immune Cell Types: This analysis examines the expression of CKAP2 in ten types of immune cells.

We delved into the role and prognostic significance of CKAP2 in GC using bulk RNA-seq data. To further substantiate the role of CKAP2 in GC, we conducted an analysis of single-cell data. Using a standardized protocol, we segmented all cells into 20 distinct clusters. Subsequent annotation categorized these cells into 10 types: T cells, natural killer (NK) cells, natural killer T (NKT) cells, plasma cells, B cells, macrophages, neutrophils, dendritic cells, epithelial cells, and plasmacytoid dendritic cells (Figure 6A,B). Figure 6C presents the marker gene information for these cell types. Additionally, we annotated eight cell clusters within the TCGA dataset and observed variations in immune cell abundance between healthy individuals and GC patients. Notably, the abundance of NK cells was significantly decreased in GC patients compared to healthy patients, whereas the abundance of neutrophils was significantly increased (Figure 6D). Finally, our analysis revealed that CKAP2 was expressed in both NK cells and neutrophils (Figure 6E).

### 2.7. Different Differentiation Features of NK Cells

**Figure 7:**
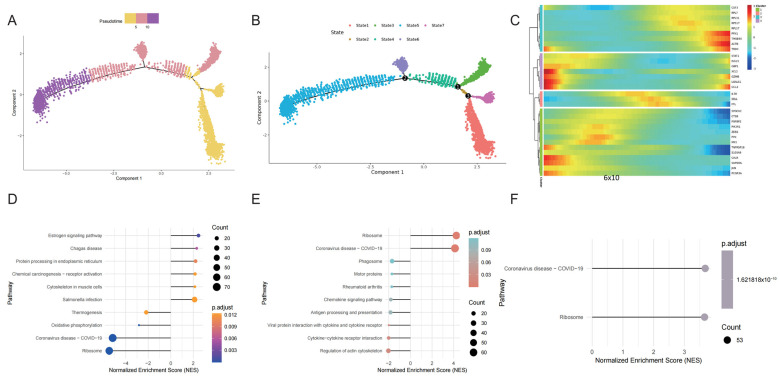

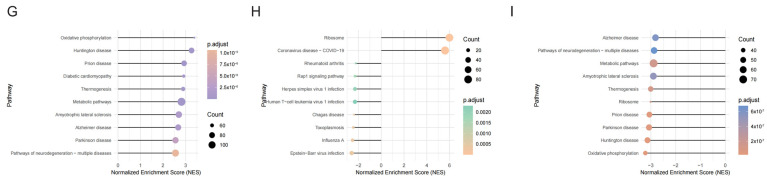
Different differentiation features of NK cells. (**A**,**B**) Seven kinds of NK cells with different differentiation were identified. (**C**) The expression of NK cell marker gene in pseudotime. (**D**–**I**) The lollipop plot of GSEA of NK cells in states 1, 3, 4, 5, 6, and 7.

CIBERSORT analysis revealed a significant disparity in the abundance of NK cells between normal individuals and those with GC. To further investigate, we utilized Monocle to analyze the pseudotime trajectory of NK cell subsets. The results indicated that NK cell subsets can exist in seven distinct states of differentiation (Figure 7A,B). A heatmap illustrating the changes in the expression of NK cell marker genes along the pseudotime trajectory is presented in Figure 7C. Subsequently, we carried out GSEA on these NK cells with different differentiation states. The results showed that state 1 was downregulated in coronavirus disease (COVID-19) and ribosome and upregulated in salmonella infection and estrogen signaling pathway (Figure 7D). State 3 is upregulated in coronavirus disease (COVID-19) and ribosomes and downregulated in the regulation of the actin cytoskeleton and cytokine–cytokine receptor interactions (Figure 7E). State 4 is downregulated in coronavirus disease (COVID-19) and ribosomes (Figure 7F). State 5 is upregulated in metabolic pathways, oxidative phosphorylation, and thermogenesis (Figure 7G). State 6 is upregulated in coronavirus disease (COVID-19) and ribosome (Figure 7H). State 7 is downregulated in metabolic pathways, oxidative phosphorylation, and thermogenesis (Figure 7I). These results show that NK cells in States 1 and 7 are in an immune suppression state, NK cells in States 3, 4, and 6 are in an immune activation state, and NK cells in State 5 are in an activation state. In addition, NK cells differentiated from immune suppression to immune activation and then differentiated into activated NK cells. In general, we identified seven kinds of NK cells with different differentiation states, and NK cells in different states have different regulatory functions in disease and related signal pathways.

### 2.8. Potential Drug Therapy

**Figure 8:**
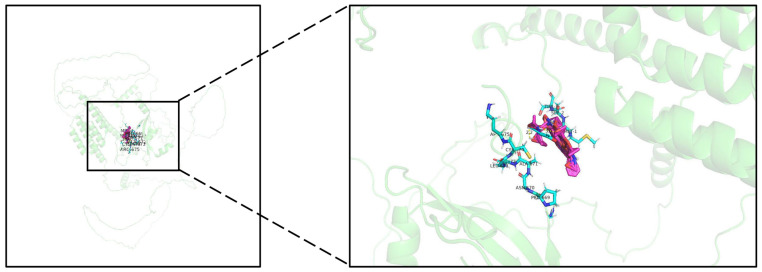
Potential drug therapy. Molecular docking of FR900359 with CKAP2.

We conducted a screening of small molecule compounds capable of enhancing CKAP2 expression and employed Autodock for molecular docking analysis. A particular small molecule compound was found to dock with CKAP2, thereby promoting its expression. The docking results of this small molecule compound with CKAP2 are depicted in Figure 8. The docking binding free energy was determined to be −10.74 kcal/mol, suggesting a stable binding interaction between the small molecule compound and the biological macromolecule.

## 3. Discussion

GC ranks among the most prevalent malignancies contributing to mortality [2]. While traditional clinicopathological parameters, like the TNM staging system, can provide some indication of the overall survival time for GC patients, their predictive accuracy is limited due to the inherent heterogeneity of tumors [19]. Adjuvant therapy is often recommended for patients with advanced GC. Nevertheless, significant variability exists in survival outcomes even among patients sharing the same TNM stage or undergoing comparable treatment protocols [20]. Consequently, the identification of biomarkers for early GC detection represents an urgent and pressing issue.

CKAP2 is a cytoskeleton-associated protein primarily implicated in the function of the cell cycle and mitosis, playing a pivotal role in the formation of intracellular microtubules and spindle bodies [21,22,23]. Studies have reported that CKAP2 shows low protein expression during the G1 phase, begins to escalate at the G1/S transition phase, and peaks during the G2/M phase [24]. Prior research has highlighted the predominant role of CKAP2 in multiple cancer types [25,26,27,28]. However, its specific function in GC remains elusive. In this study, we meticulously analyzed the expression profile of CKAP2 in GC, delving deeper into its prognostic implications, functional enrichment analysis, and immune microenvironment, with the aim of elucidating the essence of CKAP2 in GC.

Our study demonstrated that an increase in CKAP2 expression is associated with a decreased risk of GC. Furthermore, our analysis of CKAP2 expression in normal tissues and GC tissues revealed that CKAP2 is upregulated in GC compared to normal tissues, aligning with previous research findings [26]. Therefore, CKAP2 holds potential as a diagnostic biomarker for GC. Kaplan–Meier analysis further revealed that GC patients with higher CKAP2 expression exhibited a more favorable prognosis. These observations allow us to infer the function of CKAP2 in the initiation and development of GC, as well as its correlation with patient survival.

Cell proliferation stands as one crucial biological mechanism underlying tumorigenesis, rendering cell proliferation activity as a promising prognostic candidate marker [29]. The prognostic importance of cell proliferation activity has been established across various cancer types, including breast cancer [30], meningioma [31], gastrointestinal stromal tumor [32], and head and neck cancer [33]. However, its role in other cancer types remains less clear. Specifically, in the context of GC, certain studies have indicated a positive association between increased proliferative activity and lower survival rates [34], while others have failed to substantiate this relationship [35,36]. Functional enrichment analyses provided insights into the molecular mechanisms through which CKAP2 exerts its functions. Notably, the results indicated that CKAP2 and its associated genes are predominantly enriched in pathways related to the cell cycle, cellular senescence, DNA damage repair, organelle fission, nuclear division, chromosome segregation, and the p53 signaling pathway. These findings reveal that CKAP2 may play a pivotal role in GC by regulating cell cycle progression, cell proliferation, and cellular senescence.

The tumor immune microenvironment (TME) constitutes a vital aspect of tumor biology [37], comprising an intricate network of endothelial cells, fibroblasts, immune cells, and other components [38]. The interplay between cancer cells and TME constituents facilitates tumor immune escape, ultimately leading to the activation, multiplication, and infiltration of cancer cells—processes that are closely linked to tumor relapse and patient prognosis [39,40]. Our research showed that those patients with high CKAP2 expression have lower immune and stromal scores and an abundance of immune cells. These results suggest that high CKAP2 expression may reduce the need for inflammatory response and stromal remodeling by promoting self-renewal and orderly proliferation of tumor cells, leading to a decrease in the infiltration of immune cells and stromal cells. This is an important reason why high CKAP2 expression is linked with a better prognosis in GC patients. In addition, our study found that the low-expression group of CKAP2 has higher dysfunction, which indicated that the CKAP2 low-expression group had immune dysfunction. This is an important reason why high expression of CKAP2 in GC patients is related to better prognosis. The expression of CKAP2 is related to the infiltration level of various immune cells, which increases the possibility of using CKAP2 for the immunotherapy of GC.

NK cells constitute a pivotal subset of innate lymphocytes, serving as a pivotal component in the body’s immune defense against infections and cancer progression [41]. Prior research has demonstrated that diminished activity levels of NK cells in the peripheral blood are indicative of an increased risk of cancer development [42]. NK cell-based therapy has demonstrated its effectiveness in augmenting the effectiveness of tumor treatment strategies, thereby offering a promising avenue for tumor immunotherapy [43,44]. Although the molecular attributes of NK cells in various solid tumors have been well-documented, their role in the diagnosis and prognosis of GC remains largely unknown [45,46,47]. Our study reveals a notable discrepancy in the abundance of NK cells between GC patients and healthy individuals. Notably, CKAP2 is expressed in NK cells and is involved in their differentiation, thus heightening the potential for utilizing CKAP2 in GC immunotherapy.

Our research revealed a notable association between elevated CKAP2 expression and a more favorable prognosis in GC patients. Consequently, we conducted a screen of several small molecular compounds capable of upregulating CKAP2 expression, utilizing Autodock for docking analysis. The results revealed that FR900359, also known as UBO-QIC, could stably bind to CKAP2 and enhance its expression. This natural compound originates from the Ardisia crenata (vermillion root) plant and was first discovered by Japanese researchers [48]. FR900359 primarily serves as a selective antagonist, targeting the G protein alpha subunit to inhibit the G protein signaling pathways [49]. The G protein signaling cascade occupies a central position in a multitude of physiological and pathological occurrences. In recent years, the research advancements of FR900359 in the fields of cancer [49], cardiovascular diseases [50], and G protein-related diseases [51] have garnered considerable attention. Our study shows that FR900359 can stably bind to and promote the expression of the CKAP2 gene, providing insights into targeted therapy for GC. Nonetheless, developing targeted cancer treatments remains a formidable challenge. Previous studies have shown that nanomaterials [52], aptamer systems [53], and colloidal drug delivery systems [54] can effectively deliver drugs to specific target cells or organs, thereby minimizing toxicity.

This study does possess certain limitations. Primarily, our research has been conducted predominantly within the European population to mitigate population stratification bias. However, given the diversity of ethnic backgrounds, the applicability of our findings to other ancestral populations may be constrained. As we acquire additional genome-wide association study (GWAS) data on GC from diverse ancestral backgrounds, we intend to conduct supplementary research to address this issue. Furthermore, our study lacks corresponding experiments to delve into the specific mechanisms by which CKAP2 functions in GC. Consequently, the role of CKAP2 in GC necessitates further exploration through animal experiments and clinical studies. These endeavors will provide a deeper understanding of CKAP2’s involvement in GC and potentially open the door to innovative therapeutic approaches.

## 4. Materials and Methods

### 4.1. Data Collection and Analysis

**Figure 9:**
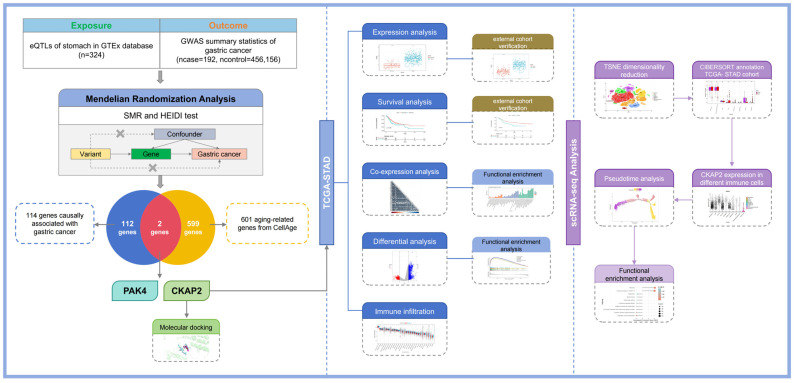
Research workflow.

We obtained genome-wide association summary statistics for 456,348 Europeans with GC, comprising 192 cases and 456,156 controls, from the GWAS Catalog (https://www.ebi.ac.uk/gwas/, accessed on 9 August 2024) [55]. We used the Version 8 release of the eQTL summarized data from the Genotype-Tissue Expression (GTEx) project [56], encompassing cis-eQTL summary data across 49 human tissues. For this study, we employed the cis-eQTL data from stomach tissue as instrumental variables for conducting SMR analysis. The aging-associated gene set, consisting of 601 genes, was derived from the CellAge database (https://genomics.senescence.info/cells/, accessed on 12 August 2024). Additionally, we downloaded GC single-cell RNA sequencing (scRNA-seq) data (GSE228598) from the Gene Expression Omnibus (GEO) database (https://www.ncbi.nlm.nih.gov/geo/, accessed on 15 August 2024). The GSE228598 dataset comprises data from 28 GC patients. Bulk RNA-seq datasets, including TCGA-STAD, GSE66229, and GSE84437, were gained from the University of California Santa Cruz (UCSC) (https://xenabrowser.net/, accessed on 17 August 2024) and the GEO database. The research workflow is illustrated in Figure 9.

### 4.2. SMR Study

SMR represents an innovative method that integrates aggregate-level data from Genome-wide association studies (GWAS) with those from expression quantitative trait loci (eQTL) studies, with the aim of identifying genes whose expression levels exhibit a correlation with complex traits, due to pleiotropic effects [57]. In our research endeavor, we employed cis-eQTL as instrumental variables, gene expression as the exposure factor, and GC as the outcome variable. The MR analysis was conducted utilizing the methodology embedded within the SMR package (https://cnsgenomics.com/software/smr, accessed on 29 August 2024). Following this, we delved into the potential linkage of the observed associations by employing the Heterogeneity In Dependent Instruments (HEIDI) test.

### 4.3. Identification of Aging-Related Genes in GC

Initially, we procured a list of 601 genes associated with aging from the CellAge database. Subsequently, utilizing SMR analysis, we identified genes that demonstrate a causal link with GC. To further refine our results, an intersection analysis was performed between the genes linked to aging and those related to GC, thereby isolating genes that are pertinent to both aging and GC. Ultimately, our analysis pinpointed two genes, CKAP2 and PAK4, as being associated with both GC and aging.

### 4.4. Differential Expression of CKAP2 and PAK4 in GC

Initially, we accessed the UCSC database for bulk RNA-seq data and clinical information pertinent to GC. Next, we examined the differential expression of CKAP2 and PAK4 between GC patients and healthy individuals. To further substantiate our findings, we validated the differential expressions CKAP2 and PAK4 in GC using an external GEO cohort. Lastly, we sourced immunohistochemical results from the HPA database (http://www.proteinatlas.org/, accessed on 1 September 2024).

### 4.5. Survival Prognosis Analysis

Using the optimal cut-off values for CKAP2 and PAK4 expression, GC patients were stratified into high and low-expression groups. Initially, we employed the Kaplan–Meier (KM) tool to compare survival differences between these two groups within the TCGA cohort. To further verify our findings, we utilized an external GEO-derived cohort to assess the correlation between CKAP2 and PAK4 expression levels and survival outcomes in GC. Additionally, we conducted a multivariate Cox regression analysis to assess the relationship between CKAP2 expression, clinical characteristics, and prognosis in GC patients. Furthermore, we delved into the association between CKAP2 expression and various clinical stages of GC. Lastly, we utilized ROC curves and a nomogram to investigate the relationship between CKAP2 expression and the survival probability, providing comprehensive insights into the prognostic value of CKAP2 in GC.

### 4.6. Analysis of Co-Expression

We utilized the Pearson correlation coefficient to reveal genes that are co-expressed with CKAP2. From the TCGA cohort, we pinpointed the top 100 genes that demonstrated the highest correlation with CKAP2. Subsequently, utilizing the “clusterProfiler” package (version 3.9.2, Taoyuan City, Taiwan, China), we performed gene ontology (GO) and Kyoto Encyclopedia of Genes and Genomes (KEGG) enrichment analyses to gain deeper insights into the functional pathways and biological processes linked to these co-expressed genes.

### 4.7. Screening of Differential Genes and Functional Analysis

Based on the median expression level of CKAP2, GC patients were stratified into high and low-expression groups. Differentially expressed genes (DEGs) analysis was then conducted using the “limma” package (version 3.54.2). The screening criteria for DEGs were set as follows: I. *p*-value < 0.05; II. absolute log2 fold change (FC) > 1. A heatmap of the DEGs was subsequently illustrated using the “pheatmap” package (version 1.0.12, Estonia). To further explore the functional roles of these DEGs, KEGG, Gene Set Enrichment Analysis (GSEA), and GO enrichment analyses were performed using the “clusterProfiler” and “ggplot2” packages (version 3.4.2, Boston, Massachusetts, USA), respectively.

### 4.8. Immune Infiltration Analysis

Firstly, GC patients were stratified into high and low-expression groups based on the CKAP2 median expression level. The immune and stromal scores for both groups were calculated using the “estimate” package (version 1.0.13, San Diego, California, USA). Subsequently, the “ssGSEA” algorithm was employed to assess the link between CKAP2 expression and the abundance of 28 types of immune cells. Following this, Pearson correlation analysis was done to quantify the relationship between CKAP2 and these 28 immune cells. Lastly, the TIDE tool (accessible at http://tide.dfci.harvard.edu/, accessed on 30 September 2024) was applied to investigate the link between CKAP2 expression and immune escape mechanisms, as well as for response prediction on immune checkpoint inhibitor (ICI) treatment.

### 4.9. Quality Control of Single Cell and Annotation of Cell Type

We employed the “Seurat” package (version 4.4.0, New York City, New York, USA) to analyze scRNA-seq data. Specifically, the “IntegrateData” function was utilized to integrate and correct data from 28 GC patients, addressing batch effects. Cell quality control was rigorously conducted based on the following criteria: I. the number of expressed genes (nFeature_RNA) ranged between 500 and 5000; II. the total number of RNA counts (nCount_RNA) was between 200 and 35,000; III. the percentage of mitochondrial genes (percentage.mt) was less than 10%. Ultimately, 63,610 cells met these criteria and were selected for further analysis. Subsequently, cell type annotation was done using the “singleR” package (version 2.6.0, Cambridge, UK) in conjunction with the CellMarker2.0 databases.

### 4.10. Annotating Cell Types in Bulk-RNA Seq Data

CIBERSORT is an algorithm designed to compute a non-negative matrix of gene expression. Leveraging the expression levels of marker genes that are unique to different cell types, we were able to ascertain the relative proportions of various cell subpopulations [58]. In this study, we employed marker genes derived from eight-cell subsets as the basis for comparing variations in cell type composition between normal individuals and GC patients.

### 4.11. Identification of NK Cell Subsets and Functional Analysis

Cells were categorized into distinct subtypes using the “singleR” package in conjunction with the CellMarker 2.0 database. Following this classification, we examined the expression of CKAP2 across these various cell types. Subsequently, we constructed the pseudotime trajectory of natural killer (NK) cells using the “Monocle” package (version 2.32.0, Cambridge, Massachusetts, USA). Pseudotime trajectory analysis, which arranges cells into trajectories featuring branching points according to a predetermined set of input genes, unveiled that distinct branches represent cell populations at unique stages of differentiation. Ultimately, we utilized GSEA to delve into the functional contributions of NK cells across their various states.

### 4.12. Drug Screened and Molecular Docking

To explore the interactions between small molecular compounds and CKAP2, we employed Autodock (Linux version 4.2, La Jolla, California, USA) for molecular docking. Initially, we curated a list of small molecules known to interact with CKAP2 from the Comparative Toxicogenomics Database available at http://ctdbase.org/, accessed on 2 October 2024. Next, we retrieved the structural data for these small molecules from the PubChem database, accessible at https://pubchem.ncbi.nlm.nih.gov/, accessed on 5 October 2024. Subsequently, we sourced the translated biomolecular structure of CKAP2 from the UniProt database, located at https://www.uniprot.org/, accessed on 8 October 2024. We then proceeded with the standard docking procedure to dock these biomolecules with the small molecular compounds. A docking-free energy of less than −5 kcal/mol was considered indicative of stable binding between the small molecular compounds and the biomacromolecules. Finally, we employed PyMol (version 2.6, New York City, USA) to visualize and interpret the docking results.

### 4.13. Statistical Analysis

Statistical analyses were conducted using R software (version 4.3.2, Auckland, New Zealand). To compare differences between various groups, a non-parametric test was employed. Additionally, the log-rank test was employed to evaluate disparities in survival probabilities across samples, with statistical significance determined by a *p*-value less than 0.05. Additionally, Pearson correlation analysis was conducted to measure correlations, with significance thresholds indicated as follows: * *p* < 0.05, ** *p* < 0.01, *** *p* < 0.001, and **** *p* < 0.0001.

## 5. Conclusions

In this study, we have, for the first time, comprehensively elucidated the intricate relationship between CKAP2 expression, clinical prognosis, and immune cell infiltration in GC. This discovery may enhance our understanding of CKAP2’s role in gastric carcinogenesis and offer fresh insights into potential therapeutic avenues for GC treatment. Our findings specifically highlight that CKAP2 is highly expressed in GC, and notably, this elevated expression is associated with a more favorable prognosis. Furthermore, CKAP2 may exert its influence on GC by modulating the cell cycle, cell proliferation, and cellular senescence. Additionally, we noted a notable correlation between CKAP2 expression and the infiltration of diverse immune cell types, suggesting a potentially pivotal role for CKAP2 in the differentiation process of NK cells. Lastly, we conducted a screen for drugs targeting CKAP2, providing fresh perspectives on targeted therapy for GC. These discoveries not only enhance our comprehension of CKAP2’s role in GC but also may open up new avenues for potential therapeutic interventions for this devastating disease.

## Data Availability

The datasets generated and analyzed during the current study are available in the GWAS Catalog, GTEx, CellAge, UCSC repository, and GEO database, including TCGA-STAD, GSE66229, GSE84437, and GSE228598.

## Supplementary Materials

The supporting information can be downloaded at: https://www.mdpi.com/article/10.3390/ijms26041557/s1.
