# Supplementary material for: Identification of CKAP2 as a Potential Target for Prevention of Gastric Cancer Progression: A Multi-Omics Study
Source: Int J Mol Sci. 2025 Feb 12;26(4):1557. doi: 10.3390/ijms26041557 (PMC11855583; doi:10.3390/ijms26041557)
Supplement: Supplementary file 1 [file ijms-26-01557-s001.zip › ijms-3436659-supplementary.pdf]

Gastric cancer and aging: CKAP2 is a promising biomarker and therapeutic target

Supplemental Material

Figure S1: Correlation between CKAP2 expression and clinical stages. (A). Stage (B). N (C). M (D). T

Table S1: Identification of causal genes related to gastric cancer by SMR.

Figure S1: Correlation between CKAP2 expression and clinical stages. (A). Stage (B). N (C). M (D). T

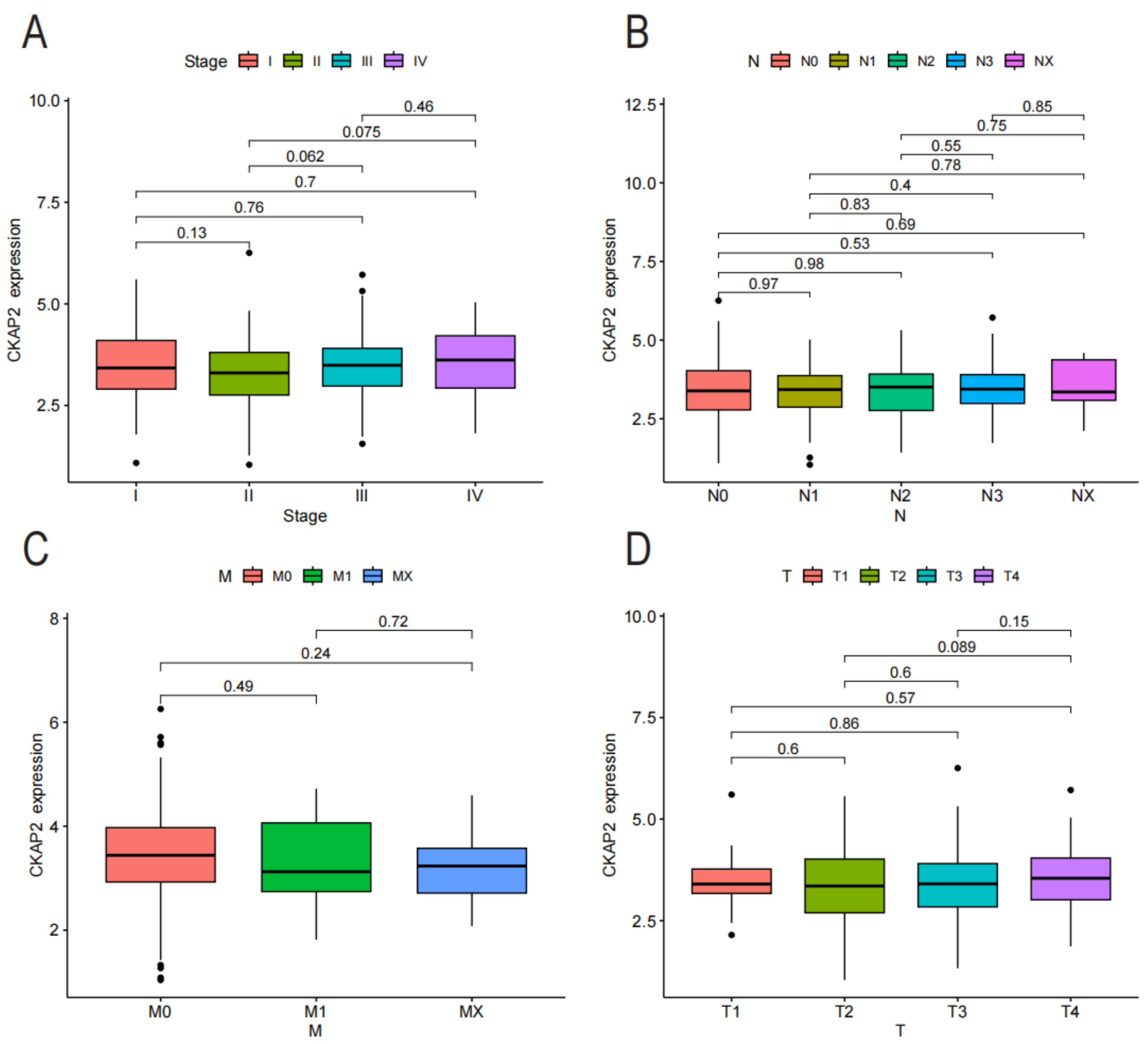

Table S1: Identification of causal genes related to gastric cancer by SMR.

	probeID	Probe Chr	Gene	Probe_bp	topSNP	topSNP _chr	topSNP_bp	A1	A2	Freq	b_GWAS	se_GWAS	p_GWAS	b_eQTL	se_eQTL	p_eQTL	b_SMR	se_SMR	p_SMR	p_HEIDI	nsnp_HEIDI	type
ENSG00000116704	ENSG00000116704	1	SLC35D1	67465015	rs12569203	1	67598347	C	A	0.531809	-0.209411	0.10291	0.0418623	-0.377975	0.0451415	5.61E-17	0.554034	0.280192	0.0480033	0.2142304	11	protein_coding
ENSG00000162688	ENSG00000162688	1	AGL	100315640	rs663848	1	100316765	T	C	0.67495	0.26782	0.111509	0.0163153	0.175882	0.0315059	2.37E-08	1.52273	0.690186	0.02736618	0.03581087	3	protein_coding
ENSG00000058085	ENSG00000058085	1	LAMC2	183155373	rs2276543	1	183155305	A	G	0.293241	-0.246045	0.114152	0.0311293	0.284286	0.0488069	5.72E-09	-0.865484	0.42815	0.04323307	0.1606264	8	protein_coding
ENSG00000143643	ENSG00000143643	1	TTC13	231041989	rs10864644	1	231108442	C	A	0.714712	0.29156	0.110496	0.00832362	-0.187654	0.0303078	5.96E-10	-1.55371	0.640069	0.01520709	0.4233023	8	protein_coding
ENSG00000169231	ENSG00000169231	1	THBS3	155165379	rs760077	1	155178782	A	T	0.394632	-0.259877	0.104235	0.0126602	-0.29638	0.0310091	1.20E-21	0.876837	0.363462	0.01584536	0.05667435	20	protein_coding
ENSG00000135773	ENSG00000135773	1	CAPN9	230883130	rs11122590	1	230903234	G	A	0.527833	0.220919	0.102341	0.0308772	0.169194	0.0240797	2.12E-12	1.30571	0.632775	0.03906785	0.1187966	14	protein_coding
ENSG00000213171	ENSG00000213171	1	LINGO4	151772740	rs12124047	1	151777927	T	C	0.0586481	0.689746	0.251456	0.00608797	1.15156	0.111171	3.83E-25	0.598967	0.225888	0.008010803	0.7019394	14	protein_coding
ENSG00000170634	ENSG00000170634	2	ACYP2	54197975	rs3731967	2	54202448	A	T	0.431412	-0.246047	0.104095	0.0180946	-0.400797	0.0467262	9.69E-18	0.613894	0.269401	0.02268247	0.000567317	20	protein_coding
ENSG00000162869	ENSG00000162869	2	PPP1R21	48667737	rs77272589	2	48681997	C	G	0.0487078	0.567072	0.228839	0.0132107	-0.492768	0.0725687	1.12E-11	-1.15079	0.494352	0.01991887	0.1455281	5	protein_coding
ENSG00000118997	ENSG00000118997	2	DNAH7	196602427	rs4622750	2	196942213	A	G	0.611332	0.269008	0.105066	0.0104564	0.80137	0.0707389	9.47E-30	0.335685	0.134415	0.01251141	0.006396237	20	protein_coding
ENSG00000196950	ENSG00000196950	2	SLC39A10	196440701	rs10208493	2	196590414	T	C	0.581511	0.429651	0.104868	4.18E-05	0.296553	0.0407858	3.57E-13	1.44882	0.405899	0.000357795	0.7297501	20	protein_coding
ENSG00000226321	ENSG00000226321	2	AC104809.3	241858202	rs11679214	2	241849938	T	C	0.477137	-0.218585	0.104072	0.0357008	-0.263107	0.0481995	4.80E-08	0.830784	0.42382	0.04996894	NA	NA	protein_coding
ENSG00000221944	ENSG00000221944	2	TIGD1	233412779	rs4973539	2	233412820	G	A	0.193837	0.394404	0.126949	0.00189125	0.252833	0.0432843	5.18E-09	1.55994	0.568709	0.00608906	0.2541261	5	protein_coding
ENSG00000162971	ENSG00000162971	2	TYW5	200794698	rs62181771	2	201042954	A	G	0.560636	-0.334801	0.104038	0.00129062	0.226298	0.0389514	6.26E-09	-1.47947	0.525555	0.004876788	0.7886893	9	protein_coding
ENSG00000115604	ENSG00000115604	2	IL18R1	102927989	rs11689730	2	102969984	A	C	0.404573	-0.221683	0.10534	0.0353388	-0.277878	0.0453595	9.00E-10	0.797771	0.400831	0.04655874	NA	NA	protein_coding
ENSG00000138468	ENSG00000138468	3	SENP7	101043049	rs10936645	3	101195898	G	A	0.411531	-0.241083	0.104304	0.0208142	-0.193147	0.032143	1.87E-09	1.24818	0.578596	0.03098509	0.07933828	4	protein_coding
ENSG00000114455	ENSG00000114455	3	HHLA2	108015376	rs1299075	3	107952752	C	A	0.558648	-0.25147	0.103202	0.0148223	0.203476	0.0265301	1.72E-14	-1.23587	0.532177	0.02021716	0.1359321	20	protein_coding
ENSG00000138459	ENSG00000138459	3	SLC35A5	112280556	rs1873571	3	112259388	G	T	0.0666004	0.505099	0.199215	0.0112307	-0.474565	0.0574221	1.40E-16	-1.06434	0.439095	0.01535306	0.5975302	8	protein_coding
ENSG00000114248	ENSG00000114248	3	LRRC31	169556967	rs2276718	3	169587741	C	G	0.128231	-0.39898	0.153979	0.00956622	-0.275861	0.0499121	3.26E-08	1.44631	0.616473	0.0189709	0.7254902	5	protein_coding
ENSG00000164081	ENSG00000164081	3	TEX264	51696709	rs11919197	3	51736344	C	T	0.874751	-0.347336	0.145315	0.0168378	-0.254604	0.0349185	3.07E-13	1.36422	0.600634	0.02312875	0.5061869	13	protein_coding
ENSG00000114857	ENSG00000114857	3	NKTR	42642106	rs627664	3	42650393	G	A	0.347913	0.282992	0.108185	0.00890166	-0.240897	0.0290999	1.25E-16	-1.17474	0.470979	0.01262216	0.7356383	20	protein_coding
ENSG00000182903	ENSG00000182903	4	ZNF721	419604	rs113830521	4	468774	A	G	0.0347913	-0.59872	0.269658	0.0263987	-0.577182	0.098887	5.32E-09	1.03732	0.499858	0.03796609	NA	NA	protein_coding
ENSG00000145246	ENSG00000145246	4	ATP10D	47487305	rs73237091	4	47543414	C	A	0.167992	0.298125	0.133686	0.0257447	0.333978	0.0518406	1.18E-10	0.892649	0.423587	0.03508655	0.2250255	3	protein_coding
ENSG00000128040	ENSG00000128040	4	SPINK2	57676026	rs115779767	4	57688007	T	G	0.0129225	1.14713	0.396382	0.00380381	0.84457	0.117011	5.28E-13	1.35824	0.505649	0.007228417	0.3308367	4	protein_coding
ENSG00000198189	ENSG00000198189	4	HSD17B11	88257762	rs6531984	4	88291535	C	T	0.728628	-0.333127	0.119602	0.00534801	0.302335	0.050132	1.63E-09	-1.10185	0.435747	0.01145058	0.2224285	6	protein_coding
ENSG00000090316	ENSG00000090316	4	MAEA	1283639	rs11721372	4	1324317	A	C	0.545726	-0.220493	0.105754	0.0370735	0.423633	0.0381493	1.19E-28	-0.520481	0.253998	0.04044694	0.9813962	20	protein_coding
ENSG00000157426	ENSG00000157426	4	AASDH	57204456	rs13134963	4	57255760	G	C	0.499006	-0.269169	0.102405	0.00857703	-0.336569	0.0414422	4.61E-16	0.799744	0.3198	0.01239266	0.915708	18	protein_coding
ENSG00000244067	ENSG00000244067	6	GSTA2	52614897	rs4715316	6	52628998	T	C	0.644135	-0.240109	0.107327	0.0252746	-0.287322	0.0400449	7.23E-13	0.835679	0.391279	0.03269892	0.268217	12	protein_coding
ENSG00000130348	ENSG00000130348	6	QRSL1	107077453	rs1026619	6	107114361	G	A	0.382704	0.209542	0.103606	0.0431253	-0.46276	0.040087	7.93E-31	-0.452809	0.227297	0.04635508	0.2064231	20	protein_coding
ENSG00000001167	ENSG00000001167	6	NFYA	41040684	rs62396296	6	41049310	T	C	0.135189	0.385318	0.165462	0.0198728	0.418045	0.0695064	1.80E-09	0.921714	0.424432	0.0298827	0.5008792	4	protein_coding
ENSG00000135318	ENSG00000135318	6	NT5E	86159809	rs6919207	6	86129271	T	C	0.100398	0.37862	0.170904	0.0267328	-0.383158	0.0499331	1.67E-14	-0.988156	0.464258	0.03329867	0.4428082	11	protein_coding
ENSG00000127952	ENSG00000127952	7	STYXL1	75625656	rs76760929	7	75625968	G	T	0.109344	0.600961	0.173941	0.000550364	0.786489	0.0688826	3.41E-30	0.764106	0.231065	0.000943377	0.001605626	15	protein_coding
ENSG00000127980	ENSG00000127980	7	PEX1	92116334	rs17687688	7	92199548	C	T	0.0357853	-0.556253	0.246986	0.0243119	1.13554	0.0883676	8.58E-38	-0.489858	0.220821	0.0265309	0.9242637	6	protein_coding
ENSG00000121716	ENSG00000121716	7	PILRB	99933737	rs113387325	7	99913146	T	G	0.173956	0.297518	0.130609	0.0227311	0.917331	0.052265	5.79E-69	0.32433	0.143573	0.02388445	0.7892124	20	protein_coding
ENSG00000085514	ENSG00000085514	7	PILRA	99965153	rs61735533	7	99955866	A	G	0.175944	0.288621	0.130018	0.0264288	1.18864	0.0663305	8.24E-72	0.242816	0.11022	0.02759365	0.8129985	20	protein_coding
ENSG00000170667	ENSG00000170667	7	RASA4B	102122892	rs17475512	7	102512488	G	A	0.12326	-0.370762	0.17157	0.030696	-0.536884	0.0908047	3.37E-09	0.690581	0.340242	0.0423896	0.1226653	10	protein_coding
ENSG00000105875	ENSG00000105875	7	WDR91	134868590	rs1646943	7	134866940	T	G	0.707753	0.267964	0.115713	0.0205712	0.468566	0.0452457	3.93E-25	0.571881	0.25305	0.02382434	0.05593906	20	protein_coding
ENSG00000198039	ENSG00000198039	7	ZNF273	64330550	rs62458435	7	64324258	C	T	0.466203	0.311605	0.103332	0.00256499	0.283105	0.040909	4.50E-12	1.10067	0.398143	0.005700878	0.1252261	20	protein_coding
ENSG00000158941	ENSG00000158941	8	CCAR2	22462145	rs2306518	8	22470308	G	A	0.371769	-0.261917	0.107222	0.0145759	0.222506	0.0287784	1.06E-14	-1.17712	0.505362	0.01984492	0.124804	16	protein_coding
ENSG00000106804	ENSG00000106804	9	C5	123714616	rs1468672	9	123809737	A	G	0.775348	0.274675	0.121594	0.0238858	0.295389	0.0477256	6.04E-10	0.929876	0.4382	0.0338349	0.3927328	5	protein_coding
ENSG00000130413	ENSG00000130413	11	STK33	8413418	rs1992756	11	8535922	A	G	0.204771	0.36446	0.136911	0.00776733	-0.444524	0.0483806	4.00E-20	-0.819888	0.320661	0.01056187	0.1598942	20	protein_coding
ENSG00000183161	ENSG00000183161	11	FANCF	22644079	rs10833793	11	22665932	C	T	0.205765	-0.2816	0.130633	0.0311102	0.235577	0.0352573	2.36E-11	-1.19536	0.582668	0.04021515	0.9254315	10	protein_coding

ENSG00000121691	ENSG00000121691	11	CAT	34460472	rs482322	11	34467490	C	T	0.290258	0.286086	0.11328	0.011554	-0.356003	0.0529516	1.78E-11	-0.803606	0.339909	0.01806994	0.3209563	12	protein_coding
ENSG00000133884	ENSG00000133884	11	DPF2	65101225	rs629710	11	65109233	C	A	0.7167	0.253453	0.113644	0.0257321	0.169413	0.0285556	2.98E-09	1.49607	0.716643	0.03683359	0.2266932	7	protein_coding
ENSG00000167325	ENSG00000167325	11	RRM1	4115937	rs11030967	11	4129120	T	C	0.583499	0.229341	0.105119	0.0291288	-0.192848	0.0287448	1.96E-11	-1.18923	0.573185	0.03800714	0.2519963	9	protein_coding
ENSG00000107537	ENSG00000107537	10	PHYH	13319796	rs12570229	10	13341185	T	C	0.699801	0.299913	0.109203	0.00602561	-0.317982	0.0422647	5.33E-14	-0.943176	0.365591	0.009883796	0.9951647	11	protein_coding
ENSG00000196932	ENSG00000196932	10	TMEM26	63166401	rs11814548	10	63223455	G	A	0.206759	-0.286537	0.136392	0.0356554	0.50482	0.0921822	4.34E-08	-0.567602	0.289378	0.04982571	0.7529787	3	protein_coding
ENSG00000165806	ENSG00000165806	10	CASP7	115438942	rs7918733	10	115439394	T	C	0.39662	0.270393	0.105992	0.0107395	0.338464	0.026681	7.11E-37	0.798883	0.319425	0.01238434	0.2670661	20	protein_coding
ENSG00000176273	ENSG00000176273	10	SLC35G1	95653730	rs10882361	10	95646730	G	A	0.450298	0.23861	0.104152	0.0219643	-0.281018	0.0462995	1.28E-09	-0.849092	0.396147	0.0320829	0.493762	3	protein_coding
ENSG00000156510	ENSG00000156510	10	HKDC1	70980059	rs4746829	10	70997888	T	C	0.4334	0.27563	0.104936	0.00862309	-0.309807	0.051867	2.33E-09	-0.889683	0.370017	0.01619743	0.01017083	6	protein_coding
ENSG00000119943	ENSG00000119943	10	PYROXD2	100143322	rs4400721	10	100147097	A	G	0.262425	-0.230639	0.113237	0.0416727	0.459585	0.0418567	4.77E-28	-0.501842	0.250593	0.04521836	0.5149749	20	protein_coding
ENSG00000069493	ENSG00000069493	12	CLEC2D	9817565	rs7967102	12	9770749	T	C	0.0815109	0.436922	0.175725	0.0129044	-0.391722	0.0683897	1.02E-08	-1.11539	0.489039	0.02256182	0.8558584	10	protein_coding
ENSG00000196458	ENSG00000196458	12	ZNF605	133498019	rs2933	12	133518838	A	G	0.217694	-0.248938	0.120798	0.0393244	0.43866	0.0467535	6.45E-21	-0.567496	0.281944	0.04413576	0.9978276	20	protein_coding
ENSG00000003056	ENSG00000003056	12	M6PR	9092959	rs4883201	12	9082581	G	A	0.11332	-0.404999	0.168842	0.0164538	-0.375816	0.0517308	3.73E-13	1.07765	0.473123	0.02274201	0.776137	6	protein_coding
ENSG00000027001	ENSG00000027001	13	MIPEP	24304328	rs11617019	13	24444557	C	T	0.133201	0.412851	0.145296	0.00449101	0.2627	0.0474191	3.03E-08	1.57157	0.621594	0.01146204	0.3854729	4	protein_coding
ENSG00000083535	ENSG00000083535	13	PIBF1	73356197	rs4885057	13	73596617	G	A	0.463221	0.270639	0.10234	0.00818109	-0.242036	0.0419911	8.22E-09	-1.11818	0.465208	0.01623427	0.3298649	11	protein_coding
ENSG00000205863	ENSG00000205863	13	C1QTNF9B	24465238	rs76106533	13	24443124	G	C	0.133201	0.414706	0.144706	0.00415877	-0.683406	0.105929	1.11E-10	-0.606822	0.231693	0.008816859	0.1071797	7	protein_coding
ENSG00000136098	ENSG00000136098	13	NEK3	52706775	rs66849828	13	52714837	A	C	0.67992	0.248143	0.10625	0.0195189	0.365061	0.0406957	2.95E-19	0.67973	0.300749	0.02381368	0.2017815	20	protein_coding
ENSG00000253710	ENSG00000253710	13	ALG11	52586534	rs1815310	13	52578240	A	T	0.601392	0.234847	0.104023	0.0239676	-0.407349	0.0499288	3.39E-16	-0.576525	0.264963	0.02956469	0.2640222	20	protein_coding
ENSG00000136108	ENSG00000136108	13	CKAP2	53029564	rs9526927	13	53051627	T	C	0.564612	0.230433	0.101875	0.0237016	-0.364284	0.0323578	2.12E-29	-0.632564	0.285247	0.02658196	0.3163215	20	protein_coding
ENSG00000165555	ENSG00000165555	14	NOXRED1	77860364	rs8018711	14	77921749	A	G	0.49006	0.282978	0.102665	0.00584549	0.357856	0.0614972	5.92E-09	0.790759	0.317446	0.01273817	0.3623001	6	protein_coding
ENSG00000151445	ENSG00000151445	14	VIPAS39	77893018	rs8005759	14	77880493	T	C	0.49006	0.296188	0.102646	0.00390778	-0.218811	0.0311149	2.03E-12	-1.35362	0.507063	0.007595525	0.3872409	4	protein_coding
ENSG00000119718	ENSG00000119718	14	EIF2B2	75469614	rs175057	14	75489632	T	C	0.461233	0.226458	0.102308	0.0268634	-0.33351	0.0353972	4.43E-21	-0.679014	0.315113	0.0311754	0.4147624	20	protein_coding
ENSG00000198805	ENSG00000198805	14	PNP	20937113	rs1760940	14	20938251	C	A	0.245527	-0.29404	0.118483	0.0130753	-0.21761	0.0258304	3.62E-17	1.35122	0.567607	0.01728609	0.6048521	8	protein_coding
ENSG00000256053	ENSG00000256053	14	APOPT1	104029299	rs861531	14	104172807	A	C	0.39662	-0.293426	0.10631	0.00577845	0.225191	0.0395113	1.20E-08	-1.30301	0.524533	0.01298682	0.01404076	18	protein_coding
ENSG00000100577	ENSG00000100577	14	GSTZ1	77787227	rs2287395	14	77791519	G	A	0.28827	0.339351	0.111176	0.00227029	0.231283	0.0305404	3.65E-14	1.46725	0.51827	0.004639355	0.9211401	20	protein_coding
ENSG00000126822	ENSG00000126822	14	PLEKHG3	65170820	rs229665	14	65171318	G	A	0.150099	-0.321903	0.149693	0.0315216	0.315757	0.0577765	4.63E-08	-1.01946	0.509456	0.04538336	NA	NA	protein_coding
ENSG00000166140	ENSG00000166140	15	ZFYVE19	41099284	rs17657877	15	41104874	C	G	0.383698	0.337505	0.107121	0.00162884	-0.224896	0.0263705	1.48E-17	-1.50072	0.507779	0.003122118	0.01686105	19	protein_coding
ENSG00000232653	ENSG00000232653	15	GOLGA8N	32885657	rs4539568	15	32902065	T	G	0.356859	0.218072	0.104429	0.0367763	-0.594154	0.0502456	2.90E-32	-0.367029	0.17848	0.03974273	0.820378	11	protein_coding
ENSG00000169371	ENSG00000169371	15	SNUPN	75890424	rs8037089	15	75921267	A	G	0.282306	0.259325	0.118392	0.0284955	0.309901	0.0447533	4.37E-12	0.836799	0.400689	0.03676147	0.5539189	12	protein_coding
ENSG00000140400	ENSG00000140400	15	MAN2C1	75648133	rs7171507	15	75737287	C	T	0.2833	0.239157	0.117498	0.0418084	0.918402	0.0486543	1.79E-79	0.260406	0.128679	0.04300285	0.02472328	20	protein_coding
ENSG00000197696	ENSG00000197696	15	NMB	85198360	rs12904605	15	85201419	C	T	0.258449	0.282407	0.115373	0.0143748	-0.471605	0.0567432	9.47E-17	-0.598821	0.255028	0.01887162	0.888971	20	protein_coding
ENSG00000206127	ENSG00000206127	15	GOLGA8O	32737307	rs1062254	15	32898631	G	C	0.353877	0.224247	0.106015	0.0344095	0.841052	0.073279	1.71E-30	0.266627	0.128173	0.03750662	0.1681955	3	protein_coding
ENSG00000177082	ENSG00000177082	15	WDR73	85185999	rs62021209	15	85203704	C	G	0.235586	0.260274	0.123527	0.0351165	-0.464716	0.0720883	1.14E-10	-0.560071	0.27965	0.04520328	0.878036	13	protein_coding
ENSG00000243789	ENSG00000243789	15	JMJD7	42120283	rs11635415	15	42133502	C	T	0.356859	-0.289877	0.108168	0.00736496	0.211271	0.038193	3.17E-08	-1.37206	0.568905	0.01587577	0.005606245	19	protein_coding
ENSG00000140265	ENSG00000140265	15	ZSCAN29	43650370	rs12906017	15	43796908	C	A	0.193837	-0.319987	0.130087	0.0139016	-0.379012	0.0571542	3.33E-11	0.844266	0.366078	0.02109691	0.7214065	19	protein_coding
ENSG00000103248	ENSG00000103248	16	MTHFSD	86563782	rs34602875	16	86566115	C	T	0.0487078	0.734148	0.252063	0.00358483	-0.953201	0.102011	9.26E-21	-0.770192	0.276987	0.005425666	0.09672046	19	protein_coding
ENSG00000103227	ENSG00000103227	16	LMF1	903634	rs112258597	16	994716	A	G	0.0934394	0.391936	0.167231	0.0190944	0.643685	0.0660494	1.93E-22	0.608894	0.26721	0.02268417	0.4642802	20	protein_coding
ENSG00000155393	ENSG00000155393	16	HEATR3	50099852	rs12926346	16	50130045	A	G	0.709742	0.223882	0.113204	0.0479644	0.639387	0.0424849	3.47E-51	0.350151	0.178573	0.04989904	0.1114801	20	protein_coding
ENSG00000135709	ENSG00000135709	16	KIAA0513	85061375	rs16975162	16	85090229	A	G	0.224652	0.284164	0.119555	0.017461	0.338004	0.0601544	1.92E-08	0.840712	0.384053	0.0285929	0.3927975	15	protein_coding
ENSG00000007171	ENSG00000007171	17	NOS2	26083792	rs3751972	17	26206414	A	C	0.757455	-0.30645	0.11786	0.00931914	-0.828724	0.0669141	3.15E-35	0.369785	0.145319	0.0109389	0.9512329	15	protein_coding
ENSG00000108406	ENSG00000108406	17	DHX40	57642886	rs9913684	17	57691502	G	A	0.238569	0.308446	0.125209	0.0137609	0.204771	0.035215	6.07E-09	1.5063	0.664066	0.02331135	0.3764624	8	protein_coding
ENSG00000108384	ENSG00000108384	17	RAD51C	56769934	rs35057477	17	57097064	A	G	0.182903	0.27633	0.123551	0.0253149	0.395211	0.0428105	2.67E-20	0.699196	0.321664	0.02972895	0.701525	20	protein_coding
ENSG00000213246	ENSG00000213246	17	SUPT4H1	56422539	rs2877877	17	56431530	G	A	0.15507	0.437626	0.136365	0.00133094	-0.246365	0.0443197	2.72E-08	-1.77633	0.639128	0.005447529	0.9483518	4	protein_coding
ENSG00000154803	ENSG00000154803	17	FLCN	17115526	rs1708618	17	17131869	C	T	0.567594	-0.217753	0.103358	0.0351362	0.800387	0.0488179	2.06E-60	-0.27206	0.130197	0.03665347	0.9953178	20	protein_coding
ENSG00000187824	ENSG00000187824	17	TMEM220	10602332	rs202308	17	10636105	C	G	0.487078	0.221765	0.103019	0.0313454	0.616369	0.0474662	1.48E-38	0.359793	0.16942	0.03369707	0.4446235	20	protein_coding
ENSG00000181396	ENSG00000181396	17	OGFOD3	80347099	rs4789769	17	80373452	T	C	0.251491	0.256232	0.126258	0.0424152	-0.385917	0.0489066	3.00E-15	-0.663956	0.33781	0.0493599	0.8959857	20	protein_coding

ENSG00000232859	ENSG00000232859	17	LYRM9	26205340	rs3751972	17	26206414	A	C	0.757455	-0.30645	0.11786	0.00931914	0.334756	0.0365212	4.91E-20	-0.915443	0.365969	0.01236948	0.8706272	11	protein_coding
ENSG00000167741	ENSG00000167741	17	GGT6	4460222	rs11657054	17	4463023	A	G	0.767396	-0.303909	0.118536	0.0103517	0.225641	0.0384864	4.55E-09	-1.34687	0.573365	0.01882071	0.9713722	8	protein_coding
ENSG00000108797	ENSG00000108797	17	CNTNAP1	40834631	rs2271029	17	40835922	C	A	0.577535	0.279858	0.105815	0.00817409	0.360972	0.0363844	3.37E-23	0.77529	0.303376	0.01060239	0.4799683	19	protein_coding
ENSG00000166750	ENSG00000166750	17	SLFN5	33570055	rs883416	17	33570441	A	C	0.500994	0.251511	0.10248	0.0141175	0.363241	0.0378247	7.74E-22	0.692408	0.291194	0.01741523	0.1943549	17	protein_coding
ENSG00000141458	ENSG00000141458	18	NPC1	21086148	rs1805081	18	21140432	C	T	0.383698	-0.22572	0.103723	0.029542	0.315946	0.0459989	6.49E-12	-0.714426	0.344377	0.03802863	0.4085398	12	protein_coding
ENSG00000198646	ENSG00000198646	20	NCOA6	33284722	rs2378257	20	33297811	C	A	0.870775	0.321206	0.132637	0.015448	-0.3277	0.0499426	5.33E-11	-0.980183	0.431438	0.02309277	0.55268	11	protein_coding
ENSG00000130684	ENSG00000130684	20	ZNF337	25654851	rs6050822	20	25694923	C	T	0.0606362	-0.531985	0.253249	0.0356723	-0.45617	0.059556	1.87E-14	1.1662	0.575663	0.04278167	0.01778273	20	protein_coding
ENSG00000124143	ENSG00000124143	20	ARHGAP40	37230577	rs6064791	20	37256636	G	A	0.294235	-0.249224	0.110945	0.0246804	-0.304281	0.0454409	2.14E-11	0.819059	0.384584	0.03319405	0.6766553	10	protein_coding
ENSG00000100987	ENSG00000100987	20	VSX1	25051521	rs2207635	20	25071504	C	T	0.228628	0.263131	0.119204	0.0272853	-0.381542	0.0636997	2.10E-09	-0.689651	0.332968	0.03833785	0.4248244	5	protein_coding
ENSG00000171940	ENSG00000171940	20	ZNF217	52183604	rs61744628	20	52186837	A	G	0.0308151	0.654826	0.312686	0.0362425	1.02541	0.101362	4.68E-24	0.638599	0.311403	0.04029451	0.6252711	3	protein_coding
ENSG00000089847	ENSG00000089847	19	ANKRD24	4183351	rs7260071	19	4180690	G	T	0.827038	0.321706	0.138271	0.0199852	0.316465	0.0569816	2.80E-08	1.01656	0.473714	0.03187803	NA	NA	protein_coding
ENSG00000197020	ENSG00000197020	19	ZNF100	21905568	rs6511291	19	21950402	C	T	0.558648	-0.276172	0.104434	0.00818187	-0.944622	0.0550834	6.40E-66	0.292362	0.111863	0.00896011	0.3192149	20	protein_coding
ENSG00000197134	ENSG00000197134	19	ZNF257	22235254	rs8105767	19	22215441	G	A	0.277336	0.30005	0.113916	0.00843968	0.65327	0.0717183	8.33E-20	0.459305	0.181522	0.01139668	0.2486659	20	protein_coding
ENSG00000237440	ENSG00000237440	19	ZNF737	20718631	rs7250879	19	20725979	C	T	0.0586481	0.621658	0.237633	0.0088954	-0.538146	0.0738168	3.09E-13	-1.15518	0.469147	0.01380449	0.6865596	3	protein_coding
ENSG00000105771	ENSG00000105771	19	SMG9	44235301	rs12669	19	44235535	A	G	0.238569	0.359356	0.125412	0.00416466	0.294555	0.0425265	4.32E-12	1.22	0.460763	0.008102357	0.05119906	6	protein_coding
ENSG00000105499	ENSG00000105499	19	PLA2G4C	48551100	rs11564512	19	48610399	G	A	0.15507	-0.294749	0.136789	0.0311794	0.279736	0.0443172	2.75E-10	-1.05367	0.5167	0.04142751	0.2691362	12	protein_coding
ENSG00000161243	ENSG00000161243	19	FBXO27	39481354	rs12983352	19	39540593	A	G	0.22664	-0.32609	0.126794	0.0101171	0.487209	0.0548702	6.73E-19	-0.669302	0.270942	0.01350082	0.1056254	14	protein_coding
ENSG00000130669	ENSG00000130669	19	PAK4	39616410	rs1529712	19	39666967	T	C	0.576541	0.233851	0.105755	0.0270184	0.156162	0.0241632	1.03E-10	1.49749	0.715756	0.03642257	0.4089913	20	protein_coding
ENSG00000189190	ENSG00000189190	19	ZNF600	53267448	rs8101566	19	53284186	G	A	0.175944	0.28554	0.12672	0.0242391	-0.641333	0.0555007	6.93E-31	-0.445229	0.20131	0.02699036	0.6799056	14	protein_coding
ENSG00000167671	ENSG00000167671	19	UBXN6	4444996	rs760369	19	4449287	C	T	0.275348	-0.265892	0.113815	0.0194823	-0.27605	0.0350042	3.12E-15	0.963202	0.430009	0.0250936	0.5964184	14	protein_coding
ENSG00000214456	ENSG00000214456	19	PLIN5	4522543	rs11881475	19	4516238	G	A	0.10338	0.451006	0.166477	0.00674611	-0.256032	0.0453581	1.65E-08	-1.76152	0.721229	0.01459044	0.2833089	10	protein_coding
ENSG00000130313	ENSG00000130313	19	PGLS	17622438	rs8104468	19	17628737	C	T	0.485089	0.30192	0.102833	0.00332458	-0.193666	0.0301714	1.37E-10	-1.55897	0.583891	0.007585747	0.3097569	7	protein_coding
ENSG00000075234	ENSG00000075234	22	TTC38	46663858	rs6008553	22	46688321	C	T	0.0248509	0.718499	0.296377	0.0153389	1.02487	0.0791194	2.25E-38	0.701064	0.294206	0.01717681	0.4772083	20	protein_coding
ENSG00000100246	ENSG00000100246	22	DNAL4	39174513	rs2294180	22	39190096	T	G	0.0805169	-0.399863	0.176002	0.0230909	-0.337388	0.049214	7.10E-12	1.18517	0.54956	0.03103816	NA	NA	protein_coding
ENSG00000214491	ENSG00000214491	22	SEC14L6	30918786	rs5753193	22	30935429	G	C	0.752485	0.272753	0.117524	0.0202961	0.606764	0.0545562	9.82E-29	0.449521	0.197862	0.0230931	0.2175339	15	protein_coding
ENSG00000160223	ENSG00000160223	21	ICOSLG	45642874	rs55708341	21	45627581	T	A	0.185885	0.405262	0.133314	0.00236647	0.844003	0.11166	4.07E-14	0.480167	0.17025	0.004796928	NA	NA	protein_coding
ENSG00000183255	ENSG00000183255	21	PTTG1IP	46269500	rs13052639	21	46273197	A	C	0.0884692	-0.429461	0.181205	0.0177868	-0.519103	0.0715099	3.89E-13	0.827314	0.367207	0.02425972	0.03635104	20	protein_coding
